# Applications of machine learning for coronary artery bypass grafting: correspondence

**DOI:** 10.1097/JS9.0000000000000052

**Published:** 2023-03-24

**Authors:** Mohammad Reza Zabihi, Mohammad Akhoondian, Narges Norouzkhani, Mohammadreza Mobayen, Pooyan Ghorbani Vajargah, Amirabbas Mollaei, Samad Karkhah

**Affiliations:** a Department of Immunology, School of Medicine, Tehran University of Medical Sciences, Tehran; b Department of Physiology, School of Medicine, Cellular and the Molecular Research Center; c Department of Medical-Surgical Nursing, School of Nursing and Midwifery; d Burn and Regenerative Medicine Research Center, Guilan University of Medical Sciences, Rasht; e Department of Medical Informatics, Faculty of Medicine, Mashhad University of Medical Sciences, Mashhad, Iran

Highlights

- The current data have shown a promising capacity for using machine learning (ML) in cardiac surgical procedures and care, especially in coronary artery bypass grafting (CABG) surgery.
- ML can be used as a platform to develop artificial intelligence-based tools with proper efficiency for other similar surgeries.
- According to present technology, the development of a prospective concept faces a significant technical challenge.
- To clarify the future direction of using ML in CABG, further studies are required to evaluate ML accuracy in CABG surgical diagnostic and therapeutic approaches.

*Dear Editor*,

Coronary artery bypass grafting (CABG) is a typical surgical procedure for treating coronary heart disease by using autologous arteries or veins as grafts to bypass coronary arteries partially or wholly obstructed by atherosclerotic plaque^1^. In this regard, many approaches were developed to improve the efficiency of the surgical procedure. However, machine learning (ML), as a part of the development of artificial intelligence, has emerged as a methodological approach that might be useful in assessing CABG risk and successes. Also, ML can be used to learn from data iteratively and identify patterns while minimizing human intervention and user input bias during or after the surgical procedure^2^. In the current study, by searching PubMed and Scopus databases, the studies that included using ML for CABG surgery were obtained, involving 23 studies. Then, the ML algorithm and assessment criteria were statistically analyzed using ‘the R programming language’ under the ggsankey, ggplot2, dplyr, and readxl libraries. According to the extracted data, the first application of ML in CABG surgery refers to 2016, and most of the studies were conducted in 2022. Also, the ‘R’ statistical analysis revealed that the ‘XGBoost’ algorithm of ML is regarded as the most used algorithm among achieved studies. In addition, the prediction of CABG surgery survival and outcome is the most targeted task of the ML tool (Fig. 1).

**Figure 1:**
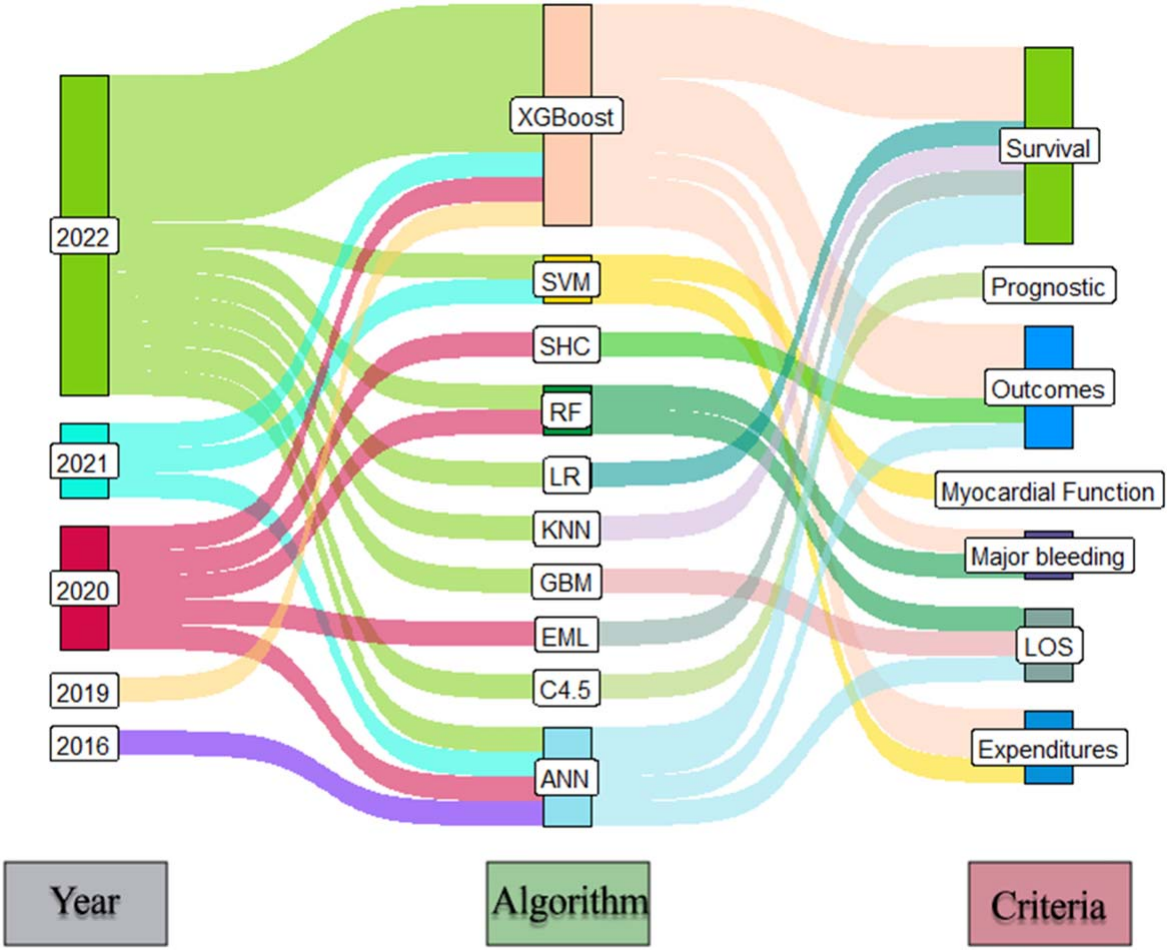
Conceptual diagram of using machine learning in coronary artery bypass grafting powered by ‘R’. ANN, artificial neural network; C4.5, decision tree algorithm; EML, ensemble machine learning; GBM, gradient-boosting machine; KNN, k-means clustering; LOS, length of stay; LR, logistic regression; RF, random forest; SHC, stochastic hill-climbing; SVM, support vector machine; XGBoost, extreme gradient boosting.

Extreme gradient boosting (XGBoost) is widely used to obtain state-of-the-art results in diverse fields with acceptable accuracy^3^. According to clinical studies, the XGBoost algorithm a has fast calculation speed and wide application in clinical data. With the assistance of the XGBoost algorithm, complex and diverse medical data can be processed to meet the requirements of timeliness and accuracy of assisted diagnosis^4^. In light of the obtained data, XGBoost can be regarded as a robust algorithm for managing and predicting CABG postoperative survival, outcomes, expenditures, and major bleeding. However, the current data revealed that the XGBoost was not used to predict criteria such as myocardial function, length of stay, or prognostic evaluations. However, the current data revealed that the XGBoost was not used to predict criteria such as myocardial function and length of stay; accordingly, the XGBoost may be considered a promising algorithm for evaluating and predicting the criteria. On the other hand, prognostic evaluations of CABG postoperatively were recognized as an emerging target for using ML in recent years. Despite their importance, capable algorithms such as XGBoost have not met prognostic criteria yet. Therefore, the postoperative prognostic evaluation can be regarded as an acceptable target for ML-related prediction tools based on algorithms such as XGBoost.

In sum, the current data have shown a promising capacity for using ML in cardiac surgical procedures and care, especially in CABG surgery. In addition, ML can be used as a platform to develop artificial intelligence-based tools with proper efficiency for other similar surgeries; however, according to the present technology, the development of a prospective concept faces a significant technical challenge^5^. Finally, to clarify the future direction of using ML in CABG, further studies are required to evaluate ML accuracy in CABG surgical diagnostic and therapeutic approaches.

## Ethical approval

This study is a type of correspondence. This article does not require any human or animal subjects to acquire such approval.

## Sources of funding

No funding.

## Author contribution

All authors involved in conceptualization, data curation, writing – original draft preparation, writing – reviewing and editing, visualization, supervision.

## Conflicts of interest disclosure

The authors declare that they have no financial conflict of interest with regard to the content of this report.

## Research registration unique identifying number (UIN)

This study is a type of correspondence and does not include human participants.

1. Name of the registry: not applicable.

2. Unique identifying number or registration ID: not applicable.

3. Hyperlink to your specific registration (must be publicly accessible and will be checked): not applicable.

## Guarantor

Samad Karkhah.

## Data statement

All data are available in the manuscript.

## Provenance and peer review

Not commissioned, internally peer-reviewed.
